# Association between emergency department utilization and the risk of child maltreatment in young children

**DOI:** 10.1186/s40621-018-0176-5

**Published:** 2018-12-20

**Authors:** Xiaoxin Kuang, Yumiko Aratani, Guohua Li

**Affiliations:** 1 Center for Injury Epidemiology and Prevention, Department of Epidemiology, Columbia University Mailman School of Public Health, 722 W 168th St, New York, NY 10032 USA; 2 Department of Health Policy and Management, Columbia University Mailman School of Public Health, 722 W 168th St, New York, NY 10032 USA; 3 Department of Anesthesiology, Columbia University College of Physicians and Surgeons, 622 W 168th St, New York, NY 10032 USA

**Keywords:** Child abuse, Child maltreatment, Emergency medical services, Injury, Electronic health records, Child welfare

## Abstract

**Background:**

This study aims to assess the association between emergency department (ED) utilization and the risk of child maltreatment.

**Methods:**

Using ED discharge data from the California’s Office of Statewide Health Planning (OSHPD) and Development for 2008–2013, we performed a nested case-control study to examine the relationship between the frequency of ED visits and child maltreatment diagnosis under 4 years of age among children born in California between 2008 and 2009 who visited the ED.

**Results:**

The study sample consisted of 3772 children diagnosed with child maltreatment (cases) and 7544 children selected by incidence density sampling (controls). After adjustment for demographic characteristics, the estimated odds ratios of child maltreatment were 1.72 (95% CI:1.55–1.90) for those with two to three ED visits and 3.03 (95% CI: 2.69–3.41) for those with four or more ED visits, compared to children with one visit. Race/ethnicity, insurance status, and location of residence were also significantly associated with the risk of child maltreatment.

**Conclusions:**

Young children with higher frequency of ED visits are at significantly increased risk of being victims of child maltreatment. ED utilization patterns and other established risk markers may assist healthcare professionals in identifying and treating victims of child maltreatment.

## Introduction

Child maltreatment, which is defined as child abuse or neglect by the World Health Organization (2016), is an important public health issue in the United States. Every year, an average of 3 million of US children are subjects of at least one investigated report of child maltreatment (U.S. Department of Health and Human Services 2017). In 2015, state agencies found an estimated 683,000 victims of child maltreatment, and approximately 1670 children died as a result of abuse and neglect (U.S. Department of Health and Human Services 2017). While child maltreatment affects all socioeconomic, ethnic, and religious groups, the risk varies with demographic and other characteristics. Compared to older children, infants and young children had consistently higher rates of victimization and maltreatment-related mortalities (Allareddy et al. 2014; Farst et al. 2013; King et al. 2015; Wu et al. 2015). Nationally, nearly 75% of child maltreatment fatalities occurred among children younger than 3 years old (U.S. Department of Health and Human Services 2017). Physical disabilities, chronic health conditions, and developmental delay can also place the child at greater risk for maltreatment (Hornor 2005; Stalker and McArthur 2012; Sullivan and Knutson 2000). Additionally, previous research has found that certain socioeconomic factors, such as household income, race, parental education, were significant predictors for child maltreatment (Brown et al. 1998; Gilbert et al. 2009; Hussey et al. 2006).

Research indicates that many victims of child maltreatment seek medical care in the hospital emergency department (ED) because of the acute nature of the injuries or a lack of primary care (King et al. 2015; Roberts et al. 2014). Emergency care may be especially important for infants and young children who are victims of maltreatment as they are more vulnerable for traumatic injuries and may require immediate medical treatment (Overpeck et al. 1998; Wu et al. 2015). Additionally, consistent evidence has shown that maltreated children had documented contact with the health care system (Guenther et al. 2009; Jenny et al. 1999; King et al. 2006; Suglia et al. 2016). Previous research found that children exposed to maltreatment and other adverse childhood experiences are more likely than other children to use medical services, in particular the ED (Guenther et al. 2009; Lanier et al. 2010; Roberts et al. 2014). However, due to the complexity of identifying child maltreatment in the clinical setting, children who arrive at the ED with severe injuries and diagnosis of physical abuse may have previously presented with less severe injuries and overlooked detection of maltreatment (Hornor 2005; Wu et al. 2015). These findings suggest that the ED setting may offer a crucial opportunity for both earlier identification and prevention for child maltreatment. In addition, young children at higher risk for maltreatment may present distinct patterns of prior ED use compared to the general pediatric population.

Despite the importance of the ED as a vital entry point for young children who experience maltreatment, the relationship between ED utilization patterns and the risk of child maltreatment diagnosis at the ED remains understudied among this population. Through a nested case-control design, the present study aimed to investigate the relationship between ED utilization and the risk of subsequent maltreatment diagnosis among a cohort of ED pediatric patients between the age of 0 and 3 in California. Our study hypothesized that young children with higher utilization of ED services are more likely to be victims of child maltreatment. The findings of the study can add to the current knowledge base of child maltreatment by characterizing readily identifiable markers for targeting high-risk children for interventions.

## Methods

### Data source

This study used the Emergency Department Discharge Data from the California’s Office of Statewide Health Planning and Development (OSHPD) for the years of 2008 to 2013. The dataset covers all ED visits in the state of California where all service records were collected from licensed hospital emergency departments on a quarterly basis. The datasets contain patient-level information, including demographic characteristics, diagnoses, treatments and procedures, as well as expected source of payment. A retrospective cohort of 259,389 children who were born between 2008 and 2009 and visited an ED in California between 2008 and 2013 was constructed based on the ED discharge data.

### Definition of child maltreatment

The evaluation of child maltreatment is often complex and difficult in the clinical settings, especially among the pediatric populations (Scott et al. 2009). To identify and classify maltreatment diagnosis for our study, we applied a comprehensive definition of maltreatment developed by King et al. (2015), which includes both explicit and suggestive maltreatment (King et al. 2015). More detailed information on the development of the maltreatment measures, including a full list of ICD-9-CM and E-codes used for both types of maltreatment, is available elsewhere (King et al. 2015). Based on the ICD-9-CM codes, the specific nature of the maltreatment can be further categorized as physical abuse, sexual abuse, and neglect. Children who experienced more than one category of maltreatment were classified as poly-victims.

### Independent variables

The main exposure of interest, ED visits during the follow-up period, was calculated with respect to the date of maltreatment diagnosis for cases. Same-day ED revisits were included in the analyses. If death occurred during an ED visit, this visit was not counted for the patient. Based on the overall frequency and distribution of ED utilization among the study population, this variable was categorized into three groups (1 visit, 2–3 visits, and ≥ 4 visits) in bivariate and multivariate analyses.

Other demographic characteristics that were evaluated in this study were sex (female vs. male), race and ethnicity (White, Black, Hispanic, Asian/Pacific Islander, and Other Race), insurance type (private insurance vs. public insurance/uninsured), and area of residence (urban vs. rural). Since information about individual income level was not available in the electronic health records, insurance status was used as a proxy to measure poverty. Specifically, children with public insurance or no insurance were considered living in poverty. The urban–rural designation for the county of patient residence was developed by the California State Office of Rural Health (California State Office of Rural Health 2012). Rural counties were identified as those with a Medical Service Study Area Rural Land Mass 80% or greater. Based on this definition, 44 out of the 58 total counties in California were considered rural while the remaining counties were classified as urban.

### Study design and population

A nested case-control study design was chosen for this study because the outcome of interest, clinical diagnosis of child maltreatment, is relatively rare. The underlying cohort from which the cases arose included children born in California between 2008 and 2009 who visited the ED from 2008 to 2013. Using incidence density sampling, cases and controls were matched on a 1:2 ratio such that for each case, two controls were randomly selected from the source population at risk at the time of case occurrence. The ratio of 1:2 matching was used in order to maximize statistical power while ensuring efficiency, as prior literature suggested that little statistical power can be gained when including more than two controls per case (Lewallen and Courtright 1998). The incidence density sampling strategy was supported by previous work that determined its ability to accurately assess the exposure effect of a time-dependent covariate and to minimize time-window bias (Di Martino et al. 2015; Richardson 2004). The first ED visit date in the study period was considered the entry visit. The follow-up period for cases was between the study entry and the date of the first maltreatment diagnosis before 4 years of age. The length of follow-up time for controls corresponded to that of their matched cases. Patients who obtained a maltreatment diagnosis on their entry ED visit were excluded from being selected as cases because there was no pattern of prior ED visits to predict the study outcome. As such, only children who obtained a maltreatment diagnosis between the age of 0 and 4 without maltreatment diagnosis on the index ED visit were used as cases in this study. All case patients were able to be matched with control patients using the statistical procedure developed by Desai, Glynn, and Gagne (Desai et al. 2016). Both cases and controls were under 4 years of age on the index date.

### Statistical analysis

To examine and understand the characteristics of the study population, a descriptive bivariate analysis was conducted for all independent predictors comparing those with a maltreatment diagnosis against those without. Chi-square test was used to assess the unadjusted statistical associations between the independent variables and the outcome measure. Because the study design involved matching between cases and controls, conditional logistic regression was used to estimate the odds ratios (ORs) and 95% confidence intervals (CIs). The incidence density sampling scheme used in a nested case-control study allowed the ORs to be a valid estimate of the incidence rate ratio. The association of ED utilization with the risk of maltreatment was evaluated through a multivariable conditional logistic regression model, while adjusting for sex, race and ethnicity, insurance status, area of residence, age at study entry (continuous in days) and stratifying by risk sets (matching cases and controls). To further evaluate the association between ED use and child maltreatment, a sensitivity analysis of the conditional logistic regression model was conducted using the sub-sample of children with one or more explicit maltreatment diagnosis. Additional models were conducted to test potential interactions between ED utilization and demographic factors. All statistical tests were conducted using SAS (version 9.4). A *p*-value of 0.05 or less was considered the indication of statistical significance in all analyses.

## Results

The study sample consisted of 3772 cases and 7544 controls. The overwhelming majority of maltreatment diagnoses at the ED were categorized as suggestive rather than explicit (Table 1). Physical abuse was the most common type of explicit maltreatment diagnosis, while neglect and sexual abuse were more commonly identified as suggestive maltreatment diagnosis. Children with a maltreatment diagnosis before 4 years of age were more likely to be non-Hispanic White or non-Hispanic African American, and less likely to be Hispanic or Asian than controls (*p* < 0.001, Table 2). Insurance type and area of residency were both influencing factors of child maltreatment (Table 2). Children who were victims of maltreatment were more likely to have public insurance (69.4% vs 61.7%, *p* < 0.001) and less likely to have private insurance (20.3% vs 27.9%, p < 0.001), compared to those without a maltreatment diagnosis. Additionally, a higher proportion of cases lived in the rural area in relative to the controls (45.2% vs 37.5%, *p* < 0.001). There were no significant differences between cases and controls with respect to gender (*p* = 0.81). Approximately 67% of cases and 51% of controls had two or more ED visits (*p* < 0.001).

**Table 1:** Characteristics and types of maltreatment diagnosis among cases of study sample (*n* = 3772), Emergency Department Discharge Data, California State Office of Statewide Health Planning and Development (OSHPD), 2008–2013

	Explicit diagnosis	Suggestive diagnosis	Total
Physical abuse	178 (73.3%)	147 (4.0%)	325 (8.3%)
Sexual abuse	29 (11.9%)	427 (11.7%)	456 (11.7%)
Neglect	10 (4.1%)	3048 (83.5%)	3058 (78.6%)
Poly-Victimization	26 (10.7%)	28 (0.77%)	54 (1.4%)
Total	243 (100.0%)	3650 (100.0%)	3893^a^

^a^The total is greater than 3772 because some cases had multiple diagnoses

**Table 2:** Emergency department (ED) utilization and demographic characteristics of the study sample by case-control status, Emergency Department Discharge Data, California State Office of Statewide Health Planning and Development (OSHPD), 2008–2013

Characteristics	Cases (*n* = 3772)		Controls (*n* = 7544)		*p*-value
	N	%	N	%	
Number of ED visits under four years of age					
1	1245	33.0	3713	49.2	< 0.001
2 to 3	1305	34.6	2470	32.7	
4 or more	1222	32.4	1361	18.0	
Sex					
Female	2033	53.9	4048	53.7	0.81
Male	1739	46.1	3496	46.3	
Race/Ethnicity					
White	1156	30.7	1686	22.4	< 0.001
Black or African American	514	13.6	859	11.4	
Hispanic	1846	48.9	4269	56.6	
Asian/Pacific Islander	97	2.6	393	5.2	
Other	159	4.2	337	4.5	
Insurance Type					
Private insurance	766	20.3	2102	27.9	< 0.001
Public insurance	2617	69.4	4654	61.7	
Uninsured	389	10.3	788	10.5	
Area of residency					
Urban	2069	54.9	4717	62.5	< 0.001
Rural	1703	45.2	2827	37.5	

In unadjusted models (Table 3), children with 2 or more ED visits were more likely to have maltreatment diagnosis at the ED than those with only one prior ED visit. In addition, living in the rural area, having public insurance, or being uninsured was also associated with an increased risk of maltreatment diagnosis at the ED. Compared to White children, those who were identified as African American, Hispanic, Asian/Pacific Islander, or other race were at decreased risk of being diagnosed with child maltreatment at the ED.

**Table 3:** Unadjusted and adjusted odds ratios and 95% confidence intervals for risk of child maltreatment by ED utilization and demographic characteristics from nested case-control analysis, Emergency Department Discharge Data, California State Office of Statewide Health Planning and Development (OSHPD), 2008–2013

Characteristics	OR (95% CI)	Adjusted OR (95% CI)
Number of ED visits under four years of age		
1 (Reference)	1.00	1.00
2 to 3	1.80 (1.63–1.99)	1.72 (1.55–1.90)
4 or more	3.37 (3.00–3.78)	3.03 (2.69–3.41)
Sex		
Female (Reference)	1.00	1.00
Male	1.01 (0.93–1.09)	1.00 (0.92–1.09)
Race/Ethnicity		
White (Reference)	1.00	1.00
Black or African American	0.86 (0.76–0.99)	0.61 (0.55–0.67)
Hispanic	0.63 (0.57–0.69)	0.85 (0.73–0.98)
Asian/Pacific Islander	0.36 (0.28–0.45)	0.46 (0.36–0.58)
Other	0.68 (0.55–0.83)	0.72 (0.58–0.89)
Insurance Type		
Private insurance (Reference)	1.00	1.00
Public insurance	1.56 (1.41–1.71)	1.36 (1.22–1.51)
Uninsured	1.36 (1.17–1.58)	1.22 (1.05–1.43)
Area of residency		
Urban (Reference)	1.00	1.00
Rural	1.36 (1.26–1.47)	1.18 (1.09–1.29)

The results of the multivariable model (Table 3) were generally consistent with those of the unadjusted models. No significant multicollinearity was found among the covariates in the multivariable regression model. The frequency of ED visits was strongly associated with risk of maltreatment diagnosis. Relative to only one visit during the follow-up period, the adjusted odds of maltreatment were 1.72 times higher (95% CI:1.55–1.90) for children with two to three visits, and 3.03 times higher (95% CI: 2.69–3.41) for those with four or more visits. Children of minority racial or ethnic groups had significantly decreased odds of being victims of maltreatment relative to White children. The adjusted odds ratios of maltreatment were 0.46 (95% CI: 0.36–0.58) for Asian and Pacific Islander, 0.61 (95% CI: 0.55–0.67) for Hispanic, 0.85 (95% CI: 0.73–0.98) for African American, and 0.72 (95% CI: 0.58–0.89) for other races. Compared to children with private insurance, children with public insurance or no insurance were shown to have 36% (95% CI: 1.22–1.51) and 22% (95% CI: 1.05–1.43) higher risk of maltreatment diagnosis, respectively. Additionally, rural residence was also associated with slightly higher odds of maltreatment diagnosis at the ED while adjusting for all other factors (adjusted OR = 1.18, 95% CI: 1.09–1.29). No significant interactions were found between ED utilization and demographic characteristics on the risk of child maltreatment in stratified analyses. In the post-hoc sensitivity analysis among the sub-sample of children with explicit maltreatment diagnosis (Table 4), the risk of being a victim of maltreatment further increased to 2.3 times (95% CI: 1.44–3.68) higher for those with two or three ED visits and 4.2 times higher for those with 4 or more ED visits (95% CI: 1.98–8.84), after controlling for other demographic covariates.

**Table 4:** Adjusted odds ratios and 95% confidence intervals for risk of child maltreatment by ED utilization and demographic characteristics from sensitivity analysis among sub-sample of children with explicit maltreatment diagnosis, Emergency Department Discharge Data, California State Office of Statewide Health Planning and Development (OSHPD), 2008–2013

Characteristics	Adjusted OR (95% CI)
Number of ED visits under four years of age	
1 (Reference)	1.00
2 to 3	2.30 (1.44–3.68)
4 or more	4.18 (1.98–8.84)
Sex	
Female (Reference)	1.00
Male	0.92 (0.61–1.38)
Race/Ethnicity^a^	
White (Reference)	1.00
Black or African American	1.29 (0.69–2.34)
Hispanic	0.54 (0.33–0.88)
Asian/Pacific Islander/Other	0.42 (0.18–0.98)
Insurance Type^a^	
Private insurance (Reference)	1.00
Public insurance/Uninsured	1.30 (0.79–2.15)
Area of residency	
Urban (Reference)	1.00
Rural	1.29 (0.85–1.97)

^a^Some subgroups were combined in the sensitivity analysis due to small sample size

## Discussion

Childhood maltreatment has been linked to increased life course risk of physical and psychological health problems in later adolescence and adulthood, including asthma, obesity, depression, alcohol and substance abuse, and violent behaviors (Gilbert et al. 2009; Hussey et al. 2006; Lanier et al. 2010). Self-reported data by adults on their adverse childhood experiences, including verbal, physical, and sexual abuse and family dysfunction, suggest that many child maltreatment victims may have not been identified during childhood (Centers for Disease and Prevention 2010). A better understanding of the risk of maltreatment and its associated factors during early childhood is critical to enhancing our ability to reduce its public health consequences and economic burdens. Our study reveals that the risk of child maltreatment increases with the frequency of ED visits in children under 4 years of age. Relative to children with one ED visit, the risk of being a victim of maltreatment increases by 72% for those with two or three ED visits and by over 200% for those with 4 or more ED visits. The results indicate that ED utilization patterns may be important risk markers of child maltreatment identified in the emergency care setting. Our findings are generally consistent with previous studies (Guenther et al. 2009; Roberts et al. 2014). In a case-control study conducted in Utah, Guenther et al. (2009) found that abused children under 13 years of age were nearly twice as likely as their peers to have had a prior ED visit (adjusted rate ratio = 1.8; 95% CI: 1.5–1.8). In a community-based cross-sectional survey involving 208 children under 6 years of age in the US Northeastern Region, Roberts et al. (2014) reported that children exposed to four or more types of traumatic events were almost three times as likely as unexposed children to report having had recently visited the ED for health purposes (adjusted OR = 2.9; 95% CI: 1.1–7.6). To further evaluate the association between ED use and child maltreatment, we conducted a post-hoc sensitivity analysis using the sub-sample of children with explicit maltreatment diagnosis. The results demonstrate an even stronger relationship between ED utilization and child maltreatment than our primary analysis which included both explicit and suggestive child maltreatment cases.

In additional to ED utilization, our study confirmed several previously identified demographic and socioeconomic factors related to child maltreatment. In the absence of information for household income, we used insurance status as a proxy to estimate individual socioeconomic status (SES). Our analysis revealed increased odds of child maltreatment diagnosis among patients who were publicly insured or had no insurance. This finding supports previous research correlating child maltreatment with markers of low SES (Allareddy et al. 2014; King et al. 2015). For instance, King et al. found that pediatric patients with an explicit maltreatment at the ED were more likely to reside in lower income neighborhoods, had public insurance or were uninsured (King et al. 2015). In another study that examined trauma outcomes among pediatric trauma patients, researchers discovered that Medicaid and uninsured children had higher mortality risk from traumatic injuries than those with private insurance (Hakmeh et al. 2010). Our study also identified significant distinctions in child maltreatment risk among different racial and ethnic groups. In particular, children from the minority groups (Black, Hispanic, Asian, Other Race) appear to have a lower risk of child maltreatment than their white peers, after controlling for all other covariates. The post-hoc sensitivity analysis, however, revealed no significant difference in the risk of explicit child maltreatment between white children and black children, while those identified as Asian, Hispanic and other race demonstrated lower risk of maltreatment compared to white children. The variability of current findings on racial disparities may owe to reliance on different data sources, such as Child Protection Services (CPS) records, hospital administrative data, and self-report survey data (Lau et al. 2003). While some studies found child maltreatment to be more common among racial minorities, especially African Americans (Drake et al. 2011; Falcone Jr. et al. 2007; Flaherty et al. 2008; Lane et al. 2002), other studies reached different conclusions (Laskey et al. 2012; Sedlak et al. 2001; Wood et al. 2010). There is an ongoing debate among researchers regarding whether sampling bias or reporting bias may have contributed to the higher incidence of child maltreatment among racial minorities reported in some studies (Ards et al. 1998; Drake et al. 2009; Jason et al. 1982; Lau et al. 2003). It is evident that African-Americans are overrepresented in the child welfare system whereas Asian and Hispanic children are underrepresented (Child Welfare Information Gateway 2016). As such, it remains unclear whether underrepresentation by Asian and Hispanic children in the child welfare system is due to cultural factors resulting in underreporting of child maltreatment and underutilization of the child welfare system or lower incidence of child maltreatment (Child Welfare Information Gateway 2016; Drake et al. 2011).

There were several notable limitations with our study. First, we examined the association between ED utilization patterns and risk of child maltreatment among children under the age of 4. As a result, our findings may not be generalizable to older children or those who have not visited the ED. Second, although we observed a significant association between ED utilization and child maltreatment, the nature of the observed relationship is unclear. We were unable to adjust for other potential confounding factors associated with child maltreatment in our analyses due to the lack of data. These include the presence of comorbidities such as chronic medical conditions or disabilities, parental education, parental mental health history, parenting style, as well as family structure. Therefore, a higher frequency of ED visits may represent a valuable risk marker for child maltreatment and a health consequence of child maltreatment, but is unlikely a causal factor for child maltreatment. Additionally, this study did not assess the severity of child maltreatment, or whether incidences of maltreatment were subsequently reported to the child protection and criminal justice systems. These unaccounted factors can be potential mechanisms that underly the association between ED utilization and child maltreatment. For instance, children who are victims of maltreatment may be more likely to have comorbidities or disabilities that result in more frequent ED visits. It is also plausible that injuries from maltreatment among young children tend to be more severe than other types of injuries and thus require immediate medical treatments. Future investigation is warranted to elicit the relationship between ED utilization and child maltreatment in order to develop effective interventions. Finally, this study identified child maltreatment based on coded discharge diagnoses from electronic health records, which might be susceptible to misclassification and under-ascertainment of child maltreatment cases, as underreporting of child maltreatment among healthcare providers is prevalent and clinical information is subject to human errors (Flaherty et al. 2008; Scott et al. 2009; Wu et al. 2015). There has been a growing recognition of importance in training health professionals to identify and document suspected maltreatment cases in the clinical setting, which requires an ongoing effort to improve and expand the current utility of ICD codes for defining maltreatment characteristics (Scott et al. 2009). In order to achieve more accurate ascertainment, future research should consider measuring and quantifying child maltreatment through linkages across multiple data sources, such as those from the healthcare, legal, and educational systems.

## Conclusion

Our results indicate that high ED utilization is an important marker for increased risk of child maltreatment. Additionally, race and ethnicity, insurance status, and area of residence were also significantly associated with child maltreatment. Identifying risk markers for child maltreatment among infants and young children is critical to the development of effective interventions in the clinical setting. In order to prevent further harm from abused victims, healthcare providers, particularly those working in the emergency care setting, play an essential role in the recognition and reporting of child maltreatment.

## Abbreviations

CI: Confidence interval
ED: Emergency department
ICD9-CM: International classification of diseases, clinical modification, ninth revision
OR: Odds ratio
OSHPD: California’s office of statewide health planning and development
US: United States
